# Cattle producers’ perceptions of biosecurity

**DOI:** 10.1186/1746-6148-9-71

**Published:** 2013-04-10

**Authors:** Marnie L Brennan, Robert M Christley

**Affiliations:** 1 Department of Epidemiology and Population Health, Institute of Infection and Global Health, Faculty of Health and Life Sciences, Leahurst Campus, University of Liverpool, Neston, CH64 7TE, UK; 2 Present address: School of Veterinary Medicine and Science, The University of Nottingham, Sutton Bonington Campus, Loughborough, LE12 5RD, UK

**Keywords:** Cattle, Cow, Cows, Bovine, Biosecurity, Prevention, Attitudes, Motivators, Barriers, Perception, Risk

## Abstract

**Background:**

The limited use of biosecurity practices by many in the farming community is likely to be due to a range of factors; further understanding of this issue is required. In this study, attitudes and behaviours of producers relating to selected biosecurity practices and the farming industry were studied by interviewing cattle farmers within a 100 km^2^ study area in north-west England using an interview-based questionnaire.

**Results:**

Most producers appeared to be familiar with the broad concept of the term biosecurity, although risks due to indirect contacts, rather than direct (animal) contacts, were more frequently highlighted. Most producers felt the nominated biosecurity practices were in some way useful, however there was not always agreement between the usefulness of a practice and it being undertaken, and vice versa. In agreement with other studies conducted in the UK, farmers most preferred to obtain information and advice on biosecurity from private veterinarians, but also highlighted DEFRA as a source.

**Conclusions:**

This study highlights the importance of understanding the motivators and barriers behind the uptake of biosecurity practices on farms, as perceptions are variable. Further understanding of these issues is needed in order to more effectively communicate information in regards to herd health and disease prevention. By identifying differences in producers’ attitudes, programs can be tailored specifically to individuals’ needs.

## Background

There are a range of benefits proposed to arise from implementation of biosecurity practices to assist in the prevention and control of disease on cattle farms. These include improved animal welfare [1], increased profit margins [2], improvement in vaccine effectiveness and reduction in incidences of antimicrobial and anthelmintic resistance [3]. In addition, consumer factors such as the demand for quality assured products [4] and public awareness of zoonoses [5] may encourage uptake of preventive practices. Enhanced job satisfaction has also been suggested as a motivator for engaging in biosecure practices [6]. It may appear prudent, therefore, for producers to use biosecurity practices to prevent or lower levels of animal disease. However, many farmers do not follow any preventive protocols and many reasons have been postulated as to why this is the case [2,7-12].

The biosecurity measures undertaken on farms appears to depend not only on economics or feasibility, but on producers’ understanding of the principles of biosecurity and their attitudes towards and motivations for undertaking/not undertaking such disease preventive measures [13]. In addition, the social network or community structure that producers belong to (i.e. what others within their peer group are doing; [14]) and the way they generally see the farming industry may also influence the undertaking of preventive measures [15-18]. The role of social behaviour and personality traits in the decision making process of producers and the subsequent effect on on-farm levels of disease or the use of preventive measures has been investigated [15,17,19-36]. Some studies have considered health behaviour theories when exploring behaviours in an animal based context. These have focused on topics such as oestrus detection [37], animal disease reporting [38], the use of biosecurity by farmers, vets and other industry partners such as hauliers [6], zoonotic disease prevention [39] and risk management strategy adoption on pig farms [40]. Further understanding of the components that effect and lead to certain behaviours, including producers’ attitudes to practices, may assist in helping to understand how to motivate and engage individuals in disease preventive activities. Advances in this area may be useful for private veterinarians and other herd health specialists looking to promote and encourage the use of preventive practices by utilising motivators or removing barriers for change.

The aims of this study were to explore producers’ understanding of the term biosecurity and producer attitudes towards recommended biosecurity practices. The relationship between these attitudes and other farm/farmer level factors, and sources of disease prevention information used by producers were also investigated to further understand the motivations of producers.

## Results

### Producers’ understanding of biosecurity and attitudes towards the farming industry

Almost all producers (88%) related the term ‘biosecurity’ to the global theme of prevention of entry of pathogens or diseases onto farms (n = 49/56; Table 1). Under a quarter of producers also described biosecurity in relation to the management of pathogens or diseases within farms (n = 15/56; 27%). Interestingly, four responses (7%) related to farm security (i.e. locking cabinets) which were not deemed to be related to the general concept of biosecurity.

**Table 1:** Themes arising from 56 farmers’ own definitions of biosecurity within the study area in North-West England

**Coding of responses**	**No. of responses contributing to theme**
**Global themes:**	
Preventing entry of diseases/pathogens onto farms	49 (88%)
Managing diseases/pathogens within farms	15 (27%)
General security (not biosecurity)	4 (7%)
Unsure	3 (5%)
**Organising themes:**	
Pathogen/disease/infection	21 (38%)
Indirect contacts between premises	21 (38%)
Within-farm management	15 (27%)
Direct contacts between premises	7 (13%)
General security (not biosecurity)	4 (7%)
Unsure	3 (5%)

More detailed understanding of biosecurity (organising themes) typically related to pathogens, diseases or infections in stock (38%, n = 21/56) and indirect contacts between farms via people, vehicles or other fomite-type transmission routes (38%, n = 21/56). Within-farm management of animals, people and equipment was the next most common theme identified (27%, n = 15/56) (Table 1).

Most farmers (64%, n = 36) believed that benefit could be attained through implementation of even a few biosecurity practices, whereas 27% (n = 15) of farmers believed that many or all practices had to be carried out for benefit to be realised (9% did not know, n = 5). Those who suggested that even a few practices could give benefit highlighted specifically concepts relating to visitors and staff cleaning and disinfecting after handling stock (n = 16/36; 44%), maintaining a closed herd (n = 8/36; 22%) and ensuring visitors and staff clean and disinfect vehicles (n = 7/36; 19%) as being beneficial. The majority of farmers believed biosecurity was more cost-effective (75%, n = 42) and more time efficient (66%, n = 37) than treating disease on-farm. Farmers were given the opportunity to nominate particular individuals that they thought should be involved in implementing and maintaining biosecurity; those highlighted included employees (n = 20/56; 36%), everyone (n = 12/56; 21%), self/wife/family (n = 10/56; 18%) and veterinarians (n = 8/56; 14%).

There were a range of opinions regarding producers’ views on the future of the UK farming industry; marginally more producers had a negative view (27%, n = 15), followed by a positive view (23%, n = 13), a very negative view (21%, n = 12) and both a positive and negative view (18%, n = 10).

### Farmers’ attitudes towards biosecurity

**Figure 1:**
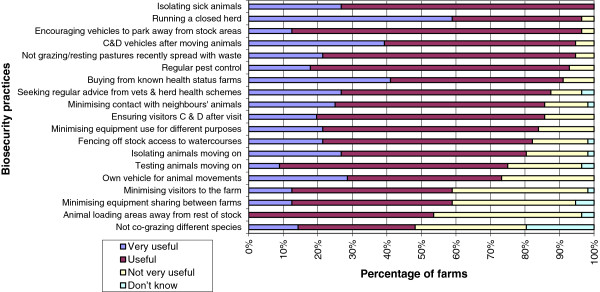
**Attitudes towards 19 biosecurity practices as nominated by 56 farmers within the study area.** C & D represents ‘clean and disinfect’.

For the majority of the 19 biosecurity practices listed, most farmers deemed them very useful or useful (Figure 1). The biosecurity practice most farmers stated as very useful was maintaining a closed herd (59%, n = 33), followed by buying animals from a farm of known disease status (41%, n = 23). The only biosecurity practice consistently nominated as being either very useful or useful was isolating sick animals. Locating animal loading areas away from where animals were situated (43%, n = 23) and minimising the number of visitors to the farm by improving security (closing gates and seeing visitors by appointment only; 39%, n = 22) were frequently nominated as being not very useful. Many farmers did not know whether not grazing different species together (20%, n = 11) or minimising the sharing of equipment and machinery with other farms (5%, n = 3) were useful or not.

Differences were seen between farmer beliefs on biosecurity (very useful/useful vs. not very useful) and practices nominated as being undertaken on farms (Table 2). The greatest differences were seen for running a closed herd and testing animals after they had been moved onto a farm; these were ranked high on the very useful/useful list, but not many farmers reported undertaking these practices. Other practices where more than 20% of farmers thought practices were useful but stated they did not carry them out were; isolating animals moving onto the farm, buying stock from known health status farms and ensuring that visitors clean and disinfect themselves after a farm visit. In addition, practices that were deemed not particularly useful but many farmers claimed to undertake them were; using your own vehicle for animal movements, minimising equipment sharing between farms and locating animal loading areas away from the rest of the stock.

**Table 2:** Biosecurity practices listed according to usefulness and whether they were nominated as being undertaken on farms by 56 farmers within the study area*

	**Very useful/useful and do**	**Very useful/useful and don’t do**	**Not very useful and do**	**Not very useful and don’t do**
Isolating sick animals	51	5	0	0
Closed herd	23	31	0	2
Encouraging vehicles to park away from stock areas	52	2	2	0
Cleaning and disinfecting vehicles after moving animals	44	0	2	0
Not grazing, or resting pastures recently spread with waste	47	2	0	3
Regular pest control	47	5	3	1
Buying from known health status farms	27	13	0	3
Seeking regular advice from vets and herd health schemes	42	7	3	2
Minimising contact with neighbours’ animals	39	9	5	2
Ensuring visitors clean and disinfect after visits	36	12	3	5
Minimising equipment use for different purposes	38	7	1	8
Fencing off stock access to watercourses	33	8	0	7
Isolating animals moving onto the farm	20	16	0	9
Testing animals moving onto the farm	3	28	0	11
Using own vehicles for animal movements	34	7	7	8
Minimising visitors to the farm	26	7	3	19
Minimising equipment sharing between farms	24	6	7	10
Locating animal loading areas away from the rest of the stock	24	6	7	17
Not co-grazing different species	2	0	1	6

*’Don’t know’ and ‘Not applicable’ responses were removed resulting in not all rows adding up to 56 responses.

### Sources of information on biosecurity

At the time of the study, most producers nominated that they sourced biosecurity information from DEFRA/government (46%, n = 26), followed by private vets (41%, n = 23) and press/farming press (18%, n = 10). Five farmers (9%) each indicated that they sourced information from farm assurance advisors, and believed biosecurity was a case of common sense/general knowledge.

**Figure 2:**
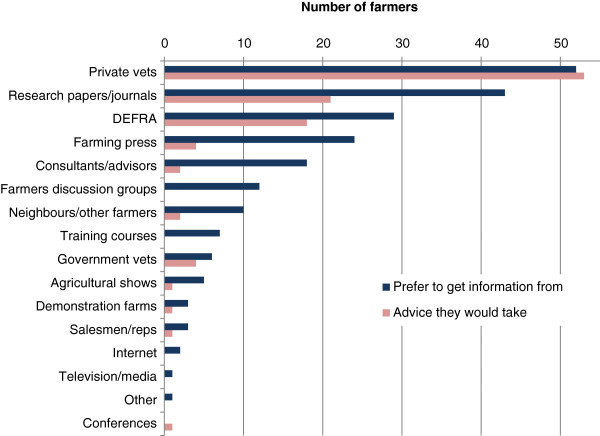
Preferred sources of information on biosecurity as nominated by 56 farmers within the study area, and whose advice farmers would be most likely to take on such issues.

The most preferred source of biosecurity information nominated from a list of providers were private vets (93%, n = 52), followed by research papers/journals (77%, n = 43) and DEFRA (52%, n = 29; Figure 2). Ten farmers (18%) responded that they would prefer to get information about biosecurity from other farmers. In terms of whose advice farmers would be most likely to take on biosecurity issues, the distribution appeared quite similar. Farmers stated that they would most likely take advice on biosecurity from private vets (95%, n = 53), followed by research papers/journals (38%, n = 21) and DEFRA (32%, n = 18); two farmers (4%) responded that they would take advice from other farmers (Figure 2).

## Discussion

There appears to be an overall understanding of the term ‘biosecurity’ in the majority of the farming community in this area, with producers having wide and varied attitudes towards the usefulness of particular biosecurity practices that have been recommended in the literature. Although this was somewhat expected, it appears the reasons behind these opinions are more varied than just farm economics and may involve other factors such as general attitudinal motivations. In order to successfully encourage the uptake of preventive practices, these motivations must be further understood [41].

In general most producers appeared to be familiar with the concept of biosecurity in broad terms in relation to the prevention of disease, although there were a proportion of respondents who appeared not to understand the usual meaning of ‘biosecurity’. Whilst the majority of farmers recognised the role of biosecurity in reduction of pathogen entry to a farm, some also linked this term with the prevention or reduction of disease spread within a farm. The term does, however, appear to be predominantly associated with the use of cleaning and disinfection to prevent transmission via indirect contacts. The emphasis on indirect transmission may be as a result of the foot and mouth disease (FMD) outbreak in the UK in 2001; after implementation of the animal movement ban, one of the practices producers could undertake to attempt to prevent the disease was the cleaning and disinfection of vehicles and people coming on to their farms; this was encouraged by the government [7] and broadly publicised. It has been postulated previously that one of the outcomes of animal epidemics is that people can be left with negative feelings towards the experience [7]; it is possible that this could affect attitudes towards actions recommended in relation to disease outbreaks in the future [42]. Farmers experience with the FMD outbreak may have had a major impact on the understanding of biosecurity at the time of this study, and may account for the results found. This also suggests that farmers’ understanding of biosecurity may not be constant and could vary over time. Although not examined here, there is some evidence that UK farmers in high bovine tuberculosis (bTB) areas view biosecurity in terms of bTB [43]. Similarly, in a study by Hernandez et al. [44], some pig producers in Australia undertook different biosecurity practices because of the perceived increased risk of disease during an outbreak. Therefore it is possible that salient diseases and experiences may drive current understanding and interpretation of terms such as biosecurity. It has been shown that FMD risks from indirect contacts can be greater than the risk from direct contacts [45], so perhaps producers in the study area were aware of this. Prevention of indirect contacts may be easier to undertake and less expensive than prevention of direct contacts (e.g. minimising the number of visitors to a farm versus buying stock from farms of known disease status), particularly if they are already being undertaken, as has been previously identified [29,46]. If producers don’t perceive something is within their capabilities, this will result in inaction. This is important when planning interventions to encourage preventive practices; farmers surrounded by other farmers who undertake risky practices in relation to disease prevention may feel less able to undertake measures themselves because they perceive they have little control over the situation (e.g. farmers not being able to alter the condition of poor boundary fences which are their neighbours’ responsibility). This was stated by several farmers during the course of the interviews, and has been demonstrated previously [47].

There can be disconnect between attitudes towards change, and the actual undertaking of actions for change. Risk aversion does not always follow on from perceived risk; farmers can perceive something as risky but can carry out the practice anyway, and vice versa [41,48-50]. In the current study, this was reflected in the finding that certain practices were perceived as useful (e.g. having a closed herd) but were not undertaken; and conversely actions perceived as not useful (e.g. locating animal loading areas away from stock) being undertaken by farmers. Farmers in a Danish study recognized the purchase of animals from dealers as risky, but some still undertook the practice [51]. This point could also be reflected by the finding that most producers believed that biosecurity was cost-effective and time-efficient, but not many stated that they carried out these practices. In contrast, there have been studies indicating that attitudes towards practices can be predictors of attitudes towards other issues [52] and even behaviours [23,53]. Further investigation is required to assess the association between attitudes and resulting actions, as the current findings suggest that different interventions may be needed depending on whether farmers think practices are useful or not. There has been research conducted in the medical and veterinary fields which address these points using health psychology models [39,54]. Knowing ‘where’ individuals are in relation to their attitudes could be utilised to facilitate action via tailored strategies [55,56].

In terms of effecting or encouraging change, understanding how producers currently find information and advice is important. It is not surprising that farmers nominated private veterinarians as preferred sources of information, and would trust their advice on which preventive practices to carry out. This has been highlighted in many other studies [6,10,23,39,44,52,57-63]. It is interesting that many farmers saw DEFRA as the main information source in relation to biosecurity in the current study. This is despite the fact that there have been previous reports of farmers generally being negative about the information they receive from DEFRA regarding biosecurity [6] and the general lack of trust individuals can have in governmental organisations [38], particularly after episodes like BSE in the UK [64]. There has been little advice published by DEFRA/AHVLA on biosecurity since this study was conducted [65]. Therefore, there may be an important role for AHVLA, or the Animal Health and Welfare Board for England (AHWBE; http://www.defra.gov.uk/ahwbe/), the new body taking forward the recommendations from the Responsibility and Cost Sharing Advisory Group, in creating advice for farmers relating to disease preventative practices as the current study indicates a need for it. It is presently not very clear what roles these organisations will have, so it is difficult to say what information resources will be available to farmers. Ultimately, behaviour change can be difficult to elicit [66], and in order to be successful, any program which aims to make a ‘cultural’ change must involve farmers in the decision-making process about how best to do this.

No information was sought to identify if attitudes of non-responders towards biosecurity differed from the participants. It is also possible that socially desirable or ‘correct’ answers may have been given by the producers in relation to their attitudes or activities, potentially leading to data bias. There is evidence in the literature to suggest that statement of behaviours from individuals and actual behaviours are not always consistent [35,67]. The top two disease-reducing practices nominated by farmers as the most useful were the most likely (and most often recommended in the literature; [68,69]) to result in the prevention of direct animal contacts. In addition, very few people nominated that they undertook these practices, which suggests that some of the answers given were not simply the socially desirable ones. Other limitations, such as how representative the farmers in this study are of other farmers within the UK, have been previously addressed in Brennan et al. [70] and Brennan and Christley [65].

## Conclusion

From the results reported here it appears that there are wide and varied attitudes towards biosecurity in the farming community; generally most farmers appear to be aware of the disease risks they are undertaking by carrying out or not carrying out certain practices, or not. However, the understanding of what the term represents and attitudinal motivations appear to have an effect on producer behaviour. In order for biosecurity to be utilised more for disease prevention, the motivations behind certain behaviours must be explored further.

## Methods

The target population was cattle farmers in a single regional area. The sampling frame was a list of cattle farmers in a 100 km^2^ study area of north-west England that had been used previously in studies conducted by the University of Liverpool [71,72]. Farmers were contacted initially by mail. Phone calls (or visits if phone numbers were not available) were then made to farmers to determine whether they were willing to participate in the study, and to obtain consent for their data to be used in the research. During discussions with farmers, 20 other potential farmers were identified that were not listed on the original list. These farmers were contacted and included in the study. Of the 81 farmers approached, 10 no longer owned cattle. Of the remaining 71, thirteen farmers declined to participate, three were shortly to cease trading, one farmer could not be contacted and one farmer couldn’t be visited in the allocated data collection period. Therefore, visits were made to 56 cattle farms and owners/managers were interviewed using a questionnaire based structure between July and September 2005, giving a response rate of 78.8% (56/71). Questions relating to direct and indirect contacts between cattle farms, and biosecurity practices undertaken on farms were asked. Results relating to some aspects of the study have been previously reported in Brennan et al. [70] and Brennan and Christley [65]. Further information relating to study design, questionnaire piloting and reasons for non-participation can be found in these publications. A copy of the questionnaire can be accessed through Brennan and Christley [65].

### Questionnaire structure relating to attitudes towards biosecurity

Using an open-interview format, farmers were asked to give their definition of biosecurity in their own words. Notes were taken by the primary author on the corresponding responses. Farmers were also asked about their thoughts on the cost-effectiveness and time-efficiency of carrying out biosecurity practices, and their general views on the farming industry (closed questions).

Closed questions were used to determine attitudes towards 19 specific biosecurity practices (see Brennan et al. [70] Appendix B for a list of practices). Farmers were asked if they thought each practice was very useful, useful or not very useful (or don’t know), as well as if they undertook any of the practices. These attitudes and whether the practices were carried out on farms were compared for discrepancies e.g. a practice was deemed by the majority of farmers to be very useful but was nominated as being undertaken infrequently.

In addition, information was gathered relating to where individuals sourced biosecurity information by asking the open question, ‘Where do you get information about biosecurity from?’. This was followed by closed questions asking ‘Where would you prefer to get information about biosecurity from?’ and ‘Whose advice would you be most likely to take about biosecurity issues?’. For these closed questions, farmers were given a checklist of sources to choose from (Table 3); time was given for farmers to read through the choices before they made their response.

**Table 3:** A list of sources of biosecurity information given to 56 farmers to choose from during on-farm interviews

Research papers/journals	Agricultural shows
Neighbours/other farmers	The internet
Demonstration farms	Consultants/advisors
Training courses	Government vets
Farmers’ discussion groups	Conferences
Farming press	Salesmen/reps
Private vets	DEFRA
Television/Media	Other (please specify)

### Data analysis

Data from the questionnaires were electronically transferred into a Microsoft Access database (Microsoft Office 2003, Microsoft Corporation) by an automated capture content system (Verity TeleForm Version 9.1; Verity Inc.). The responses to closed questions were automatically transferred by the system into the nominated database; open answer responses were entered manually.

Interpretive coding of the producers’ definitions of biosecurity was carried out. The main concepts from respondents’ definitions were classified firstly into basic themes using thematic analysis techniques [73,74]. A number of different basic theme structures were created by the first author; these themes were created after repeatedly reading the responses and identifying common elements amongst them. These basic themes were then classified into organising themes, which were subsequently organised further into global themes [73]. As farmers sometimes included several concepts in their definitions, more than 56 original concepts that were later coded were recorded.

Descriptive analyses were performed using Minitab Release 14.1 (Minitab Inc.) and SPSS 12.0.1 for Windows (SPSS Inc.).

This study was conducted in accordance with the research ethics requirements of the Faculty of Veterinary Science at the University of Liverpool. Due to the nature of the study and the low risk posed to participants, formal approval from the Ethics Committee was not a requirement at the time of the study. Potential participants were contacted by mail with information explaining the purpose and nature of the study and inviting participation. Participants were informed that their data would be anonymised, kept securely and that any material potentially leading to identification would be removed. Subsequently, potential participants were contacted by telephone (or by visiting if a phone number was not available) in order to provide further information, to obtain verbal consent and to arrange a time for the survey to be conducted. Participants were again asked to provide verbal consent prior to the interview and it was made clear that by agreeing to be interviewed, they were agreeing to be part of the study.

## Competing interests

The authors declare that they have no competing interests.

## Authors’ contributions

MB and RC conceived and designed the study. MB carried out the interviews. MB and RC analysed the data. MB and RC wrote the paper. MB and RC read and approved the final manuscript.
